# Tackling Cholera Outbreak Amidst COVID-19 Pandemic in Nigeria: Challenges and Recommendations

**DOI:** 10.3389/phrs.2022.1604776

**Published:** 2022-09-12

**Authors:** Alhaji Umar Sow, Usman Abubakar Haruna, Oladunni Abimbola Amos, Edward Olorunfemi Olajide, Terhemen Amene, Oluwatomisin Dara Odususi, Bukola Adedoyin Adewusi, Chinedu Abia, Jackson Safari, Florence Wuraola Sorinola, Hassan Olayemi Alaka, Sulaiman Muhammad Musa

**Affiliations:** ^1^ College of Medicine and Allied Health Sciences, University of Sierra Leone, Freetown, Sierra Leone; ^2^ Department of Biomedical Sciences, Nazarbayev University School of Medicine (NUSOM), Nursultan, Kazakhstan; ^3^ Faculty of Pharmaceutical Sciences, Ahmadu Bello University, Zaria, Nigeria; ^4^ Afe Babalola Multi-System Hospital, Ado-Ekiti, Nigeria; ^5^ Department of Pharmacology and Therapeutics, College of Medicine, University of Ibadan, Ibadan, Nigeria; ^6^ Faculty of Veterinary Medicine, University of Ibadan, Ibadan, Nigeria; ^7^ Faculty of Pharmacy, Igbinedion University, Okada, Nigeria; ^8^ Independent Monitoring and Evaluation, Nairobi, Kenya; ^9^ Maria Mater Misericordia Catholic Hospital, Ondo, Nigeria; ^10^ Department of Microbiology, Obafemi Awolowo University, Ile-Ife, Nigeria; ^11^ Faculty of Pharmaceutical Sciences, Ahmadu Bello University, Zaria, Nigeria

**Keywords:** COVID-19, healthcare, cholera, outbreak, WASH, Nigeria

## Abstract

**Background:** Since the first confirmed case of the Novel Coronavirus Disease 2019 (COVID-19) in Nigeria, the pandemic has become a major public health challenge, affecting different sectors of the country. While Nigeria is battling to control the spread of COVID-19, the eruption of new cholera cases has put additional pressure on the strained healthcare system.

**Evidence**: We showed how the overwhelming focus on COVID-19 has jeopardized key cholera containment measures such as disease surveillance, risk communication, and case management.

**Policy Options and Recommendations:** We recommend provision and universal access to safe water and sanitation as key cholera preventive and control measures. However, these are resources that developing countries including Nigeria find rather challenging to provide. We also proposed the implementation of well-coordinated multi-sectoral interventions that involve strengthening disease surveillance including access to safe drinking water, vaccines, and treatment, especially in vulnerable communities.

**Conclusion:** This policy brief provides evidence for policymakers, which if acted upon, will foster sustainable solutions to the lingering cholera outbreaks in Nigeria.

## Background

There are roughly 2.9 million cases of cholera each year, resulting in approximately 95,000 deaths worldwide, mainly in low and middle-income countries [1]. Since the first cholera outbreak, there has been a dramatic increase in the number of cases in African countries such as Nigeria, Sudan, and Ethiopia [2]. Throughout history, cholera outbreaks have plagued almost every continent, with developing countries being the hardest hit due to a high level of poverty. In Africa alone, 40 million people living in cholera-endemic areas are at risk of frequent outbreaks [1]. As such, cholera remains a significant public health concern, often linked to the lack of access to safe drinking water, inadequate sanitation, and poor access to the oral cholera vaccine (OCV) [1].

Coronavirus Disease 2019 (COVID-19) caused by Severe Acute Respiratory Syndrome Coronavirus 2 (SARS-COV-2) was first identified in 2019 in Hubei Province in China [3]. On the 30th of January 2020, the World Health Organization (WHO) declared COVID-19 a global public health emergency of international concern [4]. Since then, the virus has continued to spread affecting all continents across the World. Following the detection of the first case of COVID-19 in Egypt on 14th February 2020, the virus had since spread to other countries including Nigeria [5]. According to the Nigeria Center for Disease Control (NCDC), there are 248,312 confirmed cases and 3,077 deaths due to COVID-19 as of 10th January 2022 [6].

Amid the COVID-19 pandemic, seven African countries including Nigeria reported cholera outbreaks [7], which further compounded issues associated with the already strained health care systems. In Nigeria, the overwhelming focus on COVID-19 has created significant setbacks in the fight against cholera. While the government has increased awareness to strengthen prevention and control measures against cholera outbreaks [8], the needed resources are already being channeled towards the COVID-19 pandemic. Thus, creating a huge burden on the existing limited resources. In addition, the pandemic has disrupted humanitarian programs putting more pressure on the health system across the country.

This paper aimed to discuss efforts in the fight against cholera and the challenges faced in response to the cholera outbreak amidst COVID-19 in Nigeria. Thus, providing evidence for policymakers to take action and foster sustainable solutions.

## Evidence

### Efforts in the Fight Against Cholera Outbreak in Nigeria

The first series of cholera outbreaks in Nigeria was reported between 1970 and 1990 [9]. Major epidemics also occurred in 1992, 1995–1996, and 1997 [9]. Since then, the disease has become seasonal and occurs every year, mainly during the rainy season and in areas with poor sanitation.

Cholera is an acute diarrheal infection caused by the consumption of food or water contaminated with the bacterium Vibrio cholera. The WHO has highlighted the provision of safe water and sanitation as important preventive and control measures [10]. These are resources that many resources limited countries including Nigeria cannot afford or maintain. In an attempt to control the spread of cholera, the Nigerian government has continued to implement policies that involve the promotion of basic sanitation, provision of safe water, and promotion of personal hygiene practices. In addition, the government in partnership with the International Organization for Migration (IOM) supplied solar-powered boreholes, an initiative that has maintained 58 boreholes in Borno state including 11 new boreholes as of 2019 [11].

In Nigeria, the centerpiece of cholera prevention and control has been Water, Sanitation, and Hygiene (WASH) approaches, surveillance, risk communication, and case management. In 2018, National Cholera Hotspot Prioritization Survey and Risk Assessment led to the identification of 105 Local Government Areas (LGAs) as Cholera Hotspot Locations [12].

In addition, a 5 years National Strategic Plan of Action for Cholera Control was established to promote coordinated control and prevention of cholera in the country. An important component of the plan is to reduce cholera morbidity and mortality by 67% by the year 2023, which constitutes a commitment toward meeting the global target of eliminating cholera by 2030 [12]. Gearing towards these goals, the OCV administration was conducted in seven cholera hotspot locations across four like Borno, Bauchi, Yobe, and Adamawa.

In response to the sudden increase in the number of cholera cases in April 2021, the National Cholera Emergency Operations Center (EOC) was activated on the 22nd of June 2021. This led to the distribution of Rapid Response Teams to states affected by the outbreak [13]. Also, the WHO in partnership with Gavi (The Vaccine Alliance) supplied over 1.5 million doses of OCV to Bauchi state to mitigate the cholera outbreak. This led to the vaccination of 710,212 people across the state as of 28 July 2021 [14]. While OCV is an important step toward mitigating the impact of the outbreak, it does not offer a long-term solution to cholera. The government must strengthen investment in water, sanitation, and hygiene infrastructure.

### The Double Burden of Cholera and COVID19 in Nigeria

As with other countries across the world, Nigeria is trying to revamp its health system to control the third wave of the COVID-19 pandemic. However, the sudden outbreak of cholera has further worsened the fragile health system. As of December 19, 2021, a total of 109,189 suspected cholera cases and 3,604 cholera-related deaths were reported across the 32 states and the Federal Capital Territory [15]. Children between the ages of 5 and 14 appeared to be hardest hit by the cholera outbreak with a case fatality ratio of 3.3%, which is twice that associated with COVID-19. With 14% of Nigeria’s population who have access to safe drinking water, the morbidity and mortality due to cholera have significantly increased especially in the 19 northern states that account for 98% of suspected cholera cases [16]. Thus, putting millions of lives at risk of cholera-related morbidity and mortality.

While the focus is on the COVID-19 pandemic, cholera cases are silently increasing and could become a bigger threat in a country with an underserved healthcare system. This situation coupled with the interruption of humanitarian aid, inefficient human capacity [17], ongoing armed conflicts, rapid urbanization, population growth, and traditional religious beliefs [18] is likely to further weaken the country’s health system, thereby overturning progress already attained in the fight against diseases including COVID-19 and cholera if not addressed immediately.

### Current Challenges Affecting Responses to Cholera Outbreak During COVID-19 in Nigeria

#### Overburdened Healthcare System and Shortage of Health Workforce

In June 2021, following an escalating cholera outbreak across the country, the NCDC launched the National Cholera Emergency Operations Center (EOC), response techniques include the deployment of Rapid Response Teams (RRT) to help stem the outbreak at the local level, distribute medical supplies and update risk communication [13]. While the government has responded well to the outbreak, the enormous focus on COVID-19 has compounded efforts to effectively control and prevent the spread of the outbreak. Before the cholera outbreak, humanitarian response programs were suspended as a measure to reduce the spread of COVID-19. National vaccination programs are also disrupted, creating huge vaccination gaps across vulnerable communities and putting millions of lives at risk of vaccine-preventable diseases including cholera [19]. With COVID-19 stretching the healthcare system across Nigeria, there is a risk of reversal of many years of gains in the fight against cholera in the country.

The direction of the health workforce towards containing issues caused by COVID-19 has created a huge deficit of ineffective surveillance and control of cholera outbreaks. According to the World Bank, the health workforce density in Nigeria is approximately 4 doctors per 10,000 patients and 16.1 midwives and nurses per 10,000 patients [20]. This is less than the WHO’s recommendations of 1 doctor per 600 patients and a threshold level of 23 doctors, midwives, and nurses per 10,000 patients [21]. This deficit in health workforce distribution has resulted in unequal access to healthcare across communities, weighing most heavily on populations in hard-to-reach communities who appear to be the hardest hit by the cholera outbreak. With the emergence of the new omicron strain of COVID-19, coupled with the exhaustive nature of health resources and unprecedented attention towards COVID-19, Nigeria is at risk of a sudden rise in cholera cases [19].

#### Disruption of Vaccination Programs

The emergence of COVID-19 has threatened a surge in the spread and treatment of diseases endemic to Nigeria, such as cholera, Lassa fever, and meningitis, making them more transmissible and more lethal. On 20th May 2020, a total of 99 countries reported the suspension of immunization activities including oral cholera vaccines [22].

Although COVID-19 has highlighted the importance of vaccines, the current increase in cholera cases in Nigeria is due to the disruption of the cholera vaccination campaign including key cholera control components such as disease surveillance and risk communication [23]. This issue coupled with the redirection of health workers and funds towards COVID-19 has led to the disruption of health promotion activities that prevents cholera outbreaks. The disruption of the vaccines supply chain has remarkably interrupted the delivery of life-saving vaccines to some of the cholera hotspots areas in Nigeria. This has left millions of lives at risk of vaccine-preventable diseases including cholera. Hence putting more pressure on the existing weakened health system in a country known to be endemic for cholera.

#### Insufficient and Poor Access to WASH Services

In 2019, approximately 29% of Nigerians lack access to basic drinking water services, 38.1% without access to improved sanitation facilities, and 80% without access to basic handwashing facilities [24]. Additionally, in rural communities, 39% of households lack access to at least basic water supply services while only 50% have access to improved sanitation, and almost 29% practice open defecation [24]. The Nigerian government has revitalized its commitments to increasing access to WASH services in recent years, driven by the commitment to close the gap between the country and other countries in the region. This charge led to the declaration of a State of Emergency in 2018, followed by the initiation of a National Action Plan targeted at ensuring universal access to sustainable and safely managed WASH services by the year 2030 [24].

The WHO highlighted the provision of safe water and sanitation as important preventive and control measures against cholera outbreaks [4]. However, these are resources that many resources limited countries including Nigeria cannot afford or maintain. While the government has remained undeterred in the face of the outbreak by initiating cholera control measures, the situation seems to be complicated by poor access to clean water, open defecation, poor sanitation, and hygiene practices.

## Policy Options and Recommendations

Cholera remains one of the major public health challenges in Nigeria, and the recurrent outbreak has shown us the weakness of our healthcare systems. Based on available evidence and international best practices as indicated in policies of WHO, UNICEF, and CDC, the following recommendations should be adopted to mitigate the spread of the cholera outbreak in Nigeria:

### Outbreak Control and Prevention

There is a need to adopt a well-coordinated multi-sectoral approach to cholera prevention in Nigeria. This will help to identify and strengthen the connections between government agencies, bilateral and multilateral development partners, and non-governmental organizations (NGOs) whose activities help to lower the risk of cholera and promote prevention at the local and national levels. A well-coordinated cholera prevention and control strategy should be developed and implemented by the Nigerian government, and it should include robust disease surveillance as well as free and compulsory vaccination. This will aid in the early detection of cholera outbreaks, targeted prevention strategy, and partnership with state governments and non-governmental organizations (NGOs). Furthermore, the collaboration will include ministries of environment and water boards to ensure proper sanitation, sewage management, and adequate supply of clean water. If implemented, this recommendation will assist in preventing Cholera outbreaks in Nigeria, particularly during pandemics such as COVID-19. There will, however, be challenges, including funding issues, improper implementation, and corruption across various sectors.

### Rapid Case Identification and Management

While stool cultures are the most popular method for confirming cholera cases in Nigeria, rapid diagnostic test kits should be promoted once an outbreak has begun to quickly identify suspected cases, select candidate samples for stool cultures, and track epidemiological data. To achieve this, the government should empower and train the health personnel that is the closest to people such as those in community pharmacies, primary healthcare centers, and patent medicine stores to carry out rapid diagnostic tests. The recommendation may be challenged by laws governing health practices but the official Gazette granting a waiver will be sufficient to solve this problem. Therefore, the government in Nigeria should be prompt to increase the number of healthcare facilities particularly, primary and comprehensive healthcare centers at least within a reach of 1 km square. This will drastically reduce the case fatality rate (the CFR) and the spreading of the cholera infection in an outbreak.

### Water, Hygiene, and Sanitation

COVID-19 has highlighted the importance of WASH programs in controlling the spread of infectious diseases including cholera. Therefore, there is a need to strengthen policies and programs that promote WASH and waste management practices including the provision of public convenience toilets and latrines to combat open defecation. In addition, there should be at least one safe drinking water facility in every radius of 1 km^2^, especially in vulnerable communities. Also, chlorination of drinking water supplies has proven to be an effective means of preventing infectious diseases. Therefore, drinking water must be treated at the source, at points of collection or at the household level. Data on cholera surveillance must be shared with the WASH, this will timely activation of rapid response techniques.

### Health Promotion and Advocacy

There is a need to strengthen community engagement to ensure timely reports and control of cholera outbreaks at local, state, and national levels. This can be done by increasing awareness on social media and billboards in public spaces, reorienting people through the National Orientation Agency (NOA), and educating the public about disease management and prevention in local languages through radio stations. Also, advocacy should be made to the government and policy makers to provide monitoring and evaluation units to checkmate the success of the programs and compliance with the policies on tackling and preventing cholera outbreaks across Nigeria.

## Conclusion

Eliminating cholera in at least 20 countries by the year 2030 is a global target. Although Nigeria has shown commitment to achieving this goal, the sudden surge in cholera cases amidst the COVID-19 pandemic has further shown the need for preparedness and reform in the healthcare system in Nigeria. It is therefore important for policymakers to evaluate the current health system status to build resilient institutions against future disease outbreaks. There is a need for the government to take the lead in implementing coordinated multi-sectoral interventions that promote universal access to safe drinking water, sanitation, and hygiene infrastructure.
